# A Sad, Sad Case

**Published:** 1861-12

**Authors:** 


**A SAD, SAD CASE.**—A young lady, just budding into womanhood, died lately, after a ten days' illness, from pneumonia—that is, inflammation of the lungs, sometimes called lung fever or congestion of the lungs—induced by standing on the damp ground, in thin shoes, at the Central Park, having alighted from her father's carriage to look at some object of interest. It is scarcely possible that she had not been warned many times as to the danger of standing still on the damp earth, especially at certain seasons. Is there no way of leading children to heed parental advice on these and other subjects? It can not be doubted that a better result could be secured if parental counsels were followed up and corroborated by seeing them read in some journal like our own—and thus a dollar be the means of averting evils which no amount of money can remedy when once induced. Parents, think of this!

